# Aspiration thrombectomy for massive pulmonary embolism with cardiac arrest

**DOI:** 10.1093/omcr/omad093

**Published:** 2023-09-25

**Authors:** Hiroto Tamura, Shingo Kurimoto, Takaomi Harada, Shinobu Hosokawa

**Affiliations:** Department of Cardiology, Tokushima Red Cross Hospital, Tokushima, Japan; Department of Cardiology, Tokushima Red Cross Hospital, Tokushima, Japan; Department of Cardiology, Tokushima Red Cross Hospital, Tokushima, Japan; Department of Cardiology, Tokushima Red Cross Hospital, Tokushima, Japan

A 78-year-old man presented with epigastralgia and left lower-leg pain 17 days post-laparoscopic surgery for gastric cancer. Immediately after admission, he suffered cardiopulmonary arrest, and cardiopulmonary resuscitation was initiated. Return of spontaneous circulation was obtained after 20 min. An electrocardiography revealed atrial fibrillation with an S1Q3T3 pattern. Veno-arterial extracorporeal membrane oxygenation (ECMO) was initiated due to hemodynamic instability. Coronary angiography revealed no stenosis. Pulmonary angiography (PAG) revealed a massive thrombus in the right main pulmonary artery (PA) (Fig. 1A). Because of enlarging hematoma at the ECMO puncture site, thrombolysis was contraindicated. We, thus, inserted a 9-Fr 10-cm sheath through the right jugular vein and advanced a 7-Fr 80-cm sheath to the right main PA. Some thrombi were aspirated using the 7-Fr sheath with two parallel connected VacLok® syringes (Fig. 1B). Subsequently, we advanced the 7-Fr sheath to the pulmonary segmental arteries (A1, A3, A5, A8, A10), using a 6-Fr guiding catheter, and aspirated more thrombi. Although some thrombi remained, the massive thrombus was removed (Fig. 1C), and the pulmonary flow improved significantly (Fig. 1D–F). Hemodynamics improved post-procedure, and he was weaned from ECMO 3 days later. However, computed tomography revealed hypoxic encephalopathy and brainstem edema. The patient died on day 6 of hospitalization. While combined thrombolysis and percutaneous thrombectomy has been reported [1], percutaneous thrombectomy alone is also considered effective [2]. This treatment is recommended for patients with hemodynamic impairment due to acute pulmonary embolism for whom systemic thrombolysis is contraindicated or ineffective, or who may die before the effect of systemic thrombolysis [1]. We believe that more residual thrombus can be aspirated proceeding 7-Fr sheath to segmental arteries after thrombus volume reduced. Percutaneous aspiration thrombectomy can improve hemodynamics effectively and should be considered in cases where thrombolysis is contraindicated.

**Figure 1:**
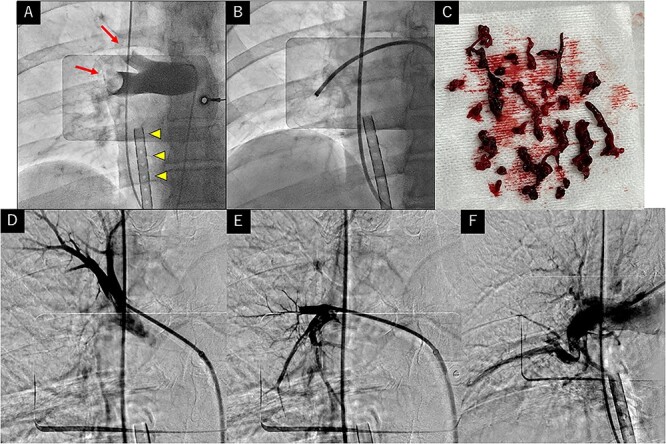
(**A**) PAG revealing massive pulmonary embolism (red arrows) and inserted veno-arterial extracorporeal membrane oxygenation (yellow arrowheads indicate the venous drainage cannula). (**B**) Thrombus aspiration performed using a 7-Fr sheath. (**C**) The removed massive thrombus. (**D**–**F**) PAG after thrombus aspiration revealing improved pulmonary flow (D) in the right A1 segment, (E) in the right A3 segment and (F) in the right basal segment of the lower lobe.

## Data Availability

All relevant data are within the manuscript.
